# Occupational physical activity and risk for prostate cancer in a nationwide cohort study in Sweden

**DOI:** 10.1038/sj.bjc.6600023

**Published:** 2002-01-07

**Authors:** A Norman, T Moradi, G Gridley, M Dosemeci, B Rydh, O Nyrén, A Wolk

**Affiliations:** Department of Medical Epidemiology, Karolinska Institutet, P.O.B. 281, SE-171 77 Stockholm, Sweden; Department of Clinical Science, Family Medicine Stockholm, Karolinska Institutet, 141 57 Huddinge, Sweden; Division of Cancer Epidemiology and Genetics, National Cancer Institute, 6120 Executive Blvd, Rockville, MD 20852-7240, USA

**Keywords:** prostatic neoplasms, occupational physical activity, exercise, cohort studies, risk factors

## Abstract

We investigated effects of occupational physical activity on relative risk for prostate cancer. From Swedish nationwide censuses in 1960 and 1970, we defined two cohorts of men whose occupational titles allowed classification of physical activity levels at work in 1960 (*n*=1 348 971) and in 1970 (*n*=1 377 629). A third cohort included only men whose jobs required a similar level of physical activity in both 1960 and 1970 (*n*=673 443). The incidence of prostate cancer between 1971 and 1989 was ascertained through record linkage to the Swedish Cancer Register. A total of 43 836, 28 702, and 19 670 prostate cancers, respectively, occurred in the three cohorts. In all three cohorts, the relative risk for prostate cancer increased with decreasing level of occupational physical activity (*P*<0.001), using Poisson regression. Among men with the same physical activity levels in 1960 and 1970, the rate ratio was 1.11 for men with sedentary jobs as compared with those whose jobs had very high/high activity levels after adjustment for age at follow-up, calendar year of follow-up and place of residence (95% CI 1.05–1.17; *P* for trend <0.001). There was no association between occupational activity and prostate cancer mortality. Since we had no data on other potential risk factors the observed associations for both incidence and mortality might have been confounded. Further studies are needed to better understand the potential role of physical activity for prostate cancer.

*British Journal of Cancer* (2002) **86**, 70–75. DOI: 10.1038/sj/bjc/6600023 www.bjcancer.com

© 2002 The Cancer Research Campaign

## 

Prostate cancer is the most common cancer among Swedish men (31.5% of all cancer cases in men in 1999 and incidence rate 159 per 100 000) and the incidence rate has been steadily increasing since the 1960s (The Swedish Cancer Registry, 2001). The causes of prostate cancer are not clearly understood and genetic and environmental/life style factors seem to be important (Ross and Schottenfeld, 1996).

Epidemiological studies of physical activity and prostate cancer risk have been inconclusive, but suggest a protective effect with higher levels of activity (Friedenreich and Thune, 2001). The theory is biologically plausible since physical activity may affect serum levels of testosterone and insulin-like growth factor I (IGF-I) and regulate the immune system (Chan *et al*, 1998b; McTiernan *et al*, 1998; Friedenreich and Thune, 2001). Moreover, physical activity might alter prostate cancer risk indirectly, by influencing body weight (Andersson *et al*, 1997). In a prospective study, both cardiorespiratory fitness and self-reported history of sports activities were inversely related to prostate cancer risk (Oliveria *et al*, 1996).

We used the nationwide, Swedish Cancer-Environment Register 60/70, to assess risk for prostate cancer in relation to occupational physical activity. We focused on men employed in 1960, and in 1970, with special attention given to men with the same level of occupational physical activity at these two assessments 10 years apart. The large number of observed prostate cancer cases permitted detailed analyses stratified by age at follow-up and by socio-economic status.

## MATERIALS AND METHODS

### Census Data

Since 1960, census information has been obtained regularly in Sweden, using questionnaires mailed to every household. The questionnaires cover demographic, occupational (including employment status, job title, industry and work address), and socio-economic factors for each household member and focus on the status during one specified week in October (Official Statistics of Sweden, 1975). Stored with the data is the national registration number (a unique personal identifier assigned to all Swedish residents) that permits linkage between registers. Since participation is mandatory by law, and since great efforts are made to trace non-responders, the censuses are more than 99% complete.

### The Cancer Register and the Cancer-Environment Register

The national Swedish Cancer Register, established in 1958, includes more than 98% of all diagnosed cancer cases in the country. It contains demographic information and detailed tumour data-site (coded according to ICD-7) and histopathology codes, date and mode of diagnosis but no information on tumour stage. The Cancer Register is linked annually to the Swedish Register of Causes of Death, which provides information on dates of death for deceased and the underlying and contributing causes of death derived from the obligatory death certificates. The proportion of prostate cancer that was histologically verified was 96–99% during the period 1971–1989.

The Cancer-Environment Register 60/70 (CER 60/70) was established by linking Cancer Register data from 1971 through 1989 to the census data from 1960 and 1970, using the national registration numbers as identifiers. The CER 60/70 proper thus includes only cancer patients who were diagnosed or died during 1971–1989 and who resided in Sweden both in 1960 and 1970 along with their census data. For comparison to the CER 60/70 proper, there is a background register with all Swedish residents who took part in both the 1960 and 1970 censuses. Except for tumour data, the information in this background register is the same as in the CER 60/70 proper, including dates (but not causes) of death for the deceased. The background register included 3 283 493 men in 1971. After record linkages, the national registration numbers were removed from both the CER 60/70 proper and the background register to ensure confidentiality. For more details about the sources of information, see Moradi *et al* (1998).

### Study cohorts

A total of 1 348 971 men in the background register reported employment in the 1960 census and had a job that we could classify with regard to physical activity. There were 1 377 629 men with such classified jobs in 1970. From the overlap of these two groups of men, we further identified the 673 443 men who had jobs requiring the same physical activity level both in 1960 and 1970 censuses.

To ascertain dates of diagnosis of prostate cancer incidence and prostate cancer as a cause of deaths in these three cohorts, we linked the background register to the CER 60/70 proper, matching on all variables that both these datasets had in common. Prostate cancers diagnosed incidentally first at autopsy were excluded from analyses. Person-years were calculated from January 1, 1971 until the diagnosis of prostate cancer, death, or end of follow-up (December 31, 1989), whichever occurred first.

### Classification of occupational physical activity and covariates

Occupations reported in the census questionnaires were coded, using a three-digit classification devised by the National Labour Market Board in Sweden, into 245 categories in the 1960 and 248 categories in the 1970 census (Official Statistics of Sweden. (1971).

Three Swedish specialists in occupational medicine, working independently, classified each occupational category as very high, high, moderate or light physical activity or sedentary. In the present analysis we considered only occupations consistently classified by the three experts, to reduce misclassification; we required agreement between at least two of the raters while the third was allowed to diverge by no more than one category (Appendix 1). A total of 202 occupations were thus unequivocally classified (Moradi *et al*, 1998). Because few men were classified as having jobs with very high physical demands, the two categories of highest physical activity (very high and high) were combined, resulting in four categories.

We used a cohort of Swedish male twins, born during 1926–1958 (Medlund *et al*, 1977), for validating the physical activity matrix. One member of each pair was randomly selected and of these, 6553 had occupations from our list and were included in our validation analysis. We found good agreement (Spearman rank correlation 0.64 *P*<0.0001) between the experts' scoring and self-reports of job-related physical activity on a 4-level scale in 1973.

Place of residence was categorized into six levels: Stockholm (capital); Gothenburg and Malmö (second and third largest cities in Sweden); other large municipalities; southern and central Sweden (except the cities and large municipalities); northern densely populated areas; northern sparsely populated areas) (Öberg and Springfeldt, 1991). We categorized socio-economic status into six levels according to Swedish classification (unskilled and semi-skilled workers, skilled workers, junior salaried employees, intermediate-level and senior salaried employees, farmers, and entrepreneurs), based on the occupational title, as described in detail elsewhere (Official Statistics of Sweden, 1995).

### Analyses

Data were analysed in grouped form. Attained age (age at follow-up) was divided into nine 5-year categories (<50, 50–54, 80–84, 85+ years). The 19 calendar years of follow-up were divided into three 5-year intervals and one 4-year (January 1, 1971 – December 31, 1975, 1976–1980, 1981–1985, 1986–1989).

We estimated the risk of prostate cancer incidence and mortality in relation to occupational physical activity by performing internal comparisons between exposure groups within the cohorts. To compute the relative risk (RR), we fitted Poisson models, in which the log Poisson rate was linear in the factors, by the maximum likelihood method (Preston *et al*, 1995). The very high/high activity category was more stable than sedentary activity category and was chosen as the reference category. The baseline model was adjusted only for age at follow-up. The second model was further adjusted for calendar year of follow-up and place of residence. We adjusted for calendar year of follow-up to take into account any differences in diagnostic routines for prostate cancer over the time period. Place of residence has been associated with prostate cancer risk in a Swedish study (Andersson *et al*, 1995). The model was further adjusted for socio-economic status. In stratified analyses, the association between physical activity and prostate cancer relative risk was investigated for different age groups (age at follow-up). Trends in relative risk were tested with the scores (high, medium, low, sedentary) equally spaced.

## RESULTS

The distribution of person-years by category of estimated occupational physical activity and the number of subjects and observed cases are shown in Table 1

**Table 1:**
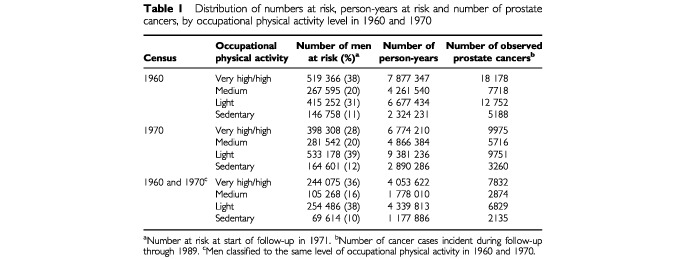
Distribution of numbers at risk, person-years at risk and number of prostate cancers, by occupational physical activity level in 1960 and 1970

. In 1960, 42% of men with classifiable jobs engaged in sedentary or light occupations, while in 1970, the corresponding proportion increased to 51%. During the 19 years of follow-up, we observed 43 836, 28 702, and 19 670 cases of prostate cancer in the three cohorts of men who had classifiable jobs in 1960, classifiable jobs in 1970, and the same physical activity level in both 1960 and 1970 censuses, respectively.

The association between the level of occupational physical activity and prostate cancer relative risk, as measured by comparisons within the cohorts, is shown in Table 2

**Table 2:**
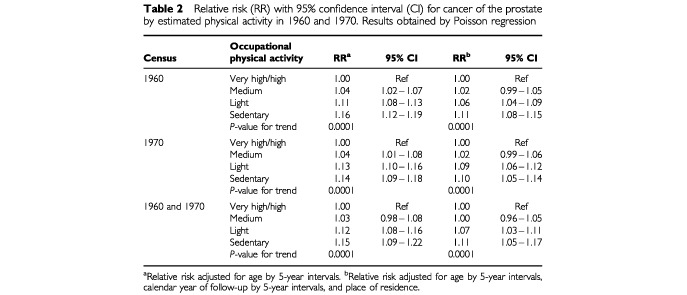
Relative risk (RR) with 95% confidence interval (CI) for cancer of the prostate by estimated physical activity in 1960 and 1970. Results obtained by Poisson regression

. In all three cohorts, prostate cancer relative risk increased slightly with decreasing level of occupational physical activity (*P*<0.001). The RR among men classified as holding sedentary jobs in both 1960 and 1970 was 11% higher than that in men estimated as having the physically most demanding jobs in 1960 and 1970. Men who worked at sedentary jobs in 1960, but changed to more physically active jobs in 1970 were still at increased risk (very high/high in 1970, RR=1.07 95% CI 0.98–1.17) compared to men with a very high/high activity level in both periods.

Occupational activity and socio-economic status are based on the same variable (job title). The Spearman correlation coefficient between occupational activity and socio-economic status was −0.67, −0.71 and −0.71 in the 1960 census, the 1970 census and in the cohort with the same level of physical activity in 1960 and 1970. Relative risk for being sedentary as compared to being very high/highly active with additional adjustment for socio-economic status was 1.09 (95% CI 1.00–1.18) in men with the same level of occupational activity in 1960 and 1970, but the model fit was not improved by adding socio-economic status. In the same model, the relative risk associated with socio-economic status was 1.06 (95% CI 1.00–1.13), when comparing senior and intermediate-level salaried employees to unskilled and semi-skilled workers.

The association between physical activity level at work and prostate cancer risk did not vary considerably by age. The relative risk for sedentary compared to very high/high was 1.09 (95% CI 1.03–1.16) for men younger than 70 years of age at follow-up, and 1.12 (95% CI 1.08–1.17) for men age 70 and over, when adjusting for age, calendar year of follow-up and place of residence.

We found no association between occupational physical activity and prostate cancer mortality. In the cohort of men with long-term occupational activity, i.e. men with the same level in both 1960 and 1970, 2638 men who died from prostate cancer during follow-up had very high/high occupational activity level, 908 men had a medium activity level, 2008 men had light activity and 605 men had a sedentary job. The relative risk for prostate cancer mortality associated with occupational activity levels of very high/high, medium, light, and sedentary was 1.00 (referent), 1.02 (0.94–1.11), 1.03 (0.96–1.10), and 1.01 (0.91–1.12) respectively, *P* for trend=0.52, when adjusting for age, calendar year of follow-up and place of residence.

## DISCUSSION

The data from our nationwide prospective cohort study supports the hypothesis that a low level of occupational physical activity might increase the relative risk of prostate cancer. Men with sedentary jobs in both 1960 and 1970 had a small, excess relative risk compared to men who had very high or high levels of activity at work. There were no significant differences in relative risk estimates for prostate cancer occurring before or after 70 years of age.

The strengths of our study are the prospective design, the nationwide population-based cohorts, the large size of the cohorts, repeated measures which indicated stable, long-term exposure, and the long follow-up time with substantial numbers of observed prostate cancer cases in all cohorts. A shortcoming is the indirect assessment of physical activity, based on job titles. However, when our physical activity matrix was validated against self-reported level of occupational physical activity in the 1970s, we found good agreement. Moreover, the use of job titles may even be an advantage, since the choice of vocation preceded the onset of the prostate cancer by many years, thus giving little room for reversed causation. Nonetheless, considerable non-differential misclassification of the factual physical activity, introduced by the crude job title classification, may have substantially attenuated the true dose-response (Rothman and Greenland, 1998). Another weakness with this study is the lack of information for individuals on possible confounders. However, there are no known strongly related risk factors for prostate cancer (Ross and Schottenfeld, 1996) and when we adjusted for place of residence no major changes in relative risk estimates were observed. Still the observed small association might be explained by unknown confounding factors.

Both occupational activity and socio-economic status are independent risk factors for prostate cancer in this data. However, since they are both based on the same variable (job title) and thus correlated, it is difficult to clearly disentangle the effect of occupational activity from the effect of socio-economic status on prostate cancer risk. The relative risk estimates for physical activity were slightly attenuated when socio-economic status was included in the model, as was also observed in another study (Dosemeci *et al*, 1993). Socio-economic status *per se* can not be biologically associated with prostate cancer, but factors closely related to social status might be and this requires closer future investigating. However, in an earlier case–control study in Sweden, socio-economic status has not been associated with prostate cancer risk (Andersson *et al*, 1996).

Ascertainment bias is a potential limitation of this study. The observed association between low occupational activity and increased prostate cancer risk could be due to the fact that manual labour is correlated with a low socio-economic status. Those men might be less likely to visit the physician and be diagnosed with prostate cancer. Regular visits to a physician in higher socio-economic groups and among older men might result in detecting latent prostate cancers among sedentary men, leading to an overestimation of relative risk for the lower activity group or among older men. However, we did not observe any significant differences in relative risk between older and younger men.

Information on total physical activity was not available in our study. Influence of recreational physical activity may be of concern if there is a systematic difference in recreational physical activity among men in different occupational activity categories. The association would be overestimated if men with sedentary jobs were less physically active during their leisure-time than men with strenuous occupations. However it is more likely that men with sedentary jobs would be more active during their leisure-time leading to an attenuation of the estimated relative risk.

Our findings are in agreement with a majority of previous studies showing a statistically significant inverse association between high level of recreational activity (Oliveria *et al*, 1996; Albanes *et al*, 1989; Hartman *et al*, 1998; Severson *et al*, 1989) and occupational activity (Brownson *et al*, 1991; Dosemeci *et al*, 1993; Hsing *et al*, 1994; Bairati *et al*, 2000; Clarke and Whittemore, 2001) and prostate cancer risk, but in contrast to two studies showing significant positive association (Ilic *et al*, 1996; Cerhan *et al*, 1997). In a follow-up study by Health Professionals, an inverse association with recreational physical activity was observed for metastatic prostate cancer only (Giovannucci *et al*, 1998). We observed a statistically significant inverse association between occupational activity and prostate cancer risk in agreement with the NHANES I study (Clarke and Whittemore, 2001), but not with the majority of other cohort studies investigating the role of occupational activity and not showing significant associations (Vena *et al*, 1987; Albanes *et al*, 1989; Severson *et al*, 1989; Thune and Lund, 1994; Steenland *et al*, 1995; Hartman *et al*, 1998; Lund Nilsen *et al*, 2000; Putnam *et al*, 2000). This may be explained by that our cohort study is the largest performed to date, thus it has enough power to show even weak associations to be statistically significant. In a case–control study estimating lifetime occupational physical activity, the increased risk for prostate cancer was higher when a larger proportion of life was spent in sedentary jobs, which is consistent with our findings (Bairati *et al*, 2000). Strenuous occupational activities in the mid-teens or early twenties have been reported to be inversely associated with prostate cancer risk (Villeneuve *et al*, 1999). These findings may indicate that physical activity in different periods in life, or the cumulative life-long occupational activity is of importance for prostate cancer risk. The lack of significant inverse association with prostate cancer mortality observed in our study does not exclude a possibility of a weak association reported previously from a large US cohort (Vena *et al*, 1987). Effect of potential confounding factors on our risk estimates might mask the true association of physical activity with prostate cancer mortality.

The association between prostate cancer and physical activity can be explained by at least three different possible mechanisms. First, high levels of bioavailable testosterone has been associated with increased risk of prostate cancer (Gann *et al*, 1996; Wolk *et al*, 1997). Several studies have reported lower levels of free testosterone (Tikkanen *et al*, 1998; Tymchuk *et al*, 2001) in athletically trained men relative to untrained men and immediately after exercise (Nindl *et al*, 2001) as well as increased levels of sex hormone binding globuline (SHBG) immediately after exercise in older men (Zmuda *et al*, 1996) and after an exercise programme (Caballero *et al*, 1992; Tymchuk *et al*, 1998). However, there are also studies showing increasing levels of testosterone with increasing levels of exercise (Zmuda *et al*, 1996). Secondly, a higher level of circulating insulin-like growth factor I (IGF-I) has been associated with increased risk of prostate cancer both in prospective (Chan *et al*, 1998a; Stattin *et al*, 2000) and in case–control studies (Wolk *et al*, 1998). IGF-I might also be associated with long-term or short-term physical activity levels, although observed associations are inconsistent (Bonnefoy *et al*, 1998; Eliakim *et al*, 1998; Tissandier *et al*, 2001). Thirdly, the immune system, which is involved in regulating the susceptibility to both the initiation and promotion of tumours, can be modified by physical activity (Shephard and Shek, 1995). Depending on intensity, duration and frequency of activity, immune functions can be suppressed or enhanced by physical activity, but moderate physical activity usually stimulates the immune response.

In summary, we have shown that work in sedentary and light physical activity occupations might be associated with increased risk of prostate cancer incidence but not mortality. The observed associations may have been confounded by factors related to socio-economic status. To better disentangle the effects of occupational physical activity from socio-economic status, future studies should include specific information on physical activity at work and during leisure-time, as well as detailed information on potential confounders.

## Figures and Tables

**Appendix 1:**
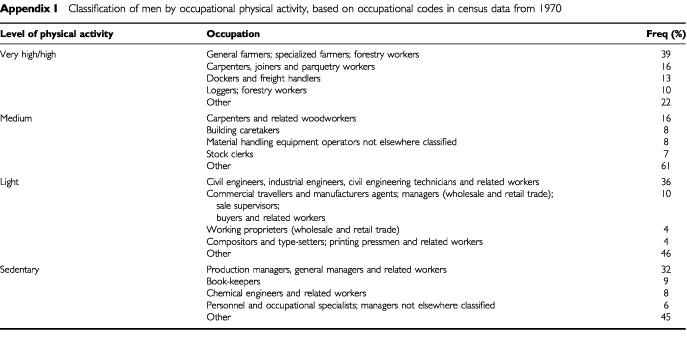
Classification of men by occupational physical activity, based on occupational codes in census data from 1970

## Appendix

Appendix 1
